# Potential Prognostic Immune Biomarkers of Overall Survival in Ovarian Cancer Through Comprehensive Bioinformatics Analysis: A Novel Artificial Intelligence Survival Prediction System

**DOI:** 10.3389/fmed.2021.587496

**Published:** 2021-05-24

**Authors:** Tingshan He, Liwen Huang, Jing Li, Peng Wang, Zhiqiao Zhang

**Affiliations:** Department of Infectious Diseases, Shunde Hospital, Southern Medical University, Guangzhou, China

**Keywords:** ovarian cancer, overall survival, immune gene, transcription factor, prognostic signature

## Abstract

**Background:** The tumour immune microenvironment plays an important role in the biological mechanisms of tumorigenesis and progression. Artificial intelligence medicine studies based on big data and advanced algorithms are helpful for improving the accuracy of prediction models of tumour prognosis. The current research aims to explore potential prognostic immune biomarkers and develop a predictive model for the overall survival of ovarian cancer (OC) based on artificial intelligence algorithms.

**Methods:** Differential expression analyses were performed between normal tissues and tumour tissues. Potential prognostic biomarkers were identified using univariate Cox regression. An immune regulatory network was constructed of prognostic immune genes and their highly related transcription factors. Multivariate Cox regression was used to identify potential independent prognostic immune factors and develop a prognostic model for ovarian cancer patients. Three artificial intelligence algorithms, random survival forest, multitask logistic regression, and Cox survival regression, were used to develop a novel artificial intelligence survival prediction system.

**Results:** The current study identified 1,307 differentially expressed genes and 337 differentially expressed immune genes between tumour samples and normal samples. Further univariate Cox regression identified 84 prognostic immune gene biomarkers for ovarian cancer patients in the model dataset (GSE32062 dataset and GSE53963 dataset). An immune regulatory network was constructed involving 63 immune genes and 5 transcription factors. Fourteen immune genes (PSMB9, FOXJ1, IFT57, MAL, ANXA4, CTSH, SCRN1, MIF, LTBR, CTSD, KIFAP3, PSMB8, HSPA5, and LTN1) were recognised as independent risk factors by multivariate Cox analyses. Kaplan-Meier survival curves showed that these 14 prognostic immune genes were closely related to the prognosis of ovarian cancer patients. A prognostic nomogram was developed by using these 14 prognostic immune genes. The concordance indexes were 0.760, 0.733, and 0.765 for 1-, 3-, and 5-year overall survival, respectively. This prognostic model could differentiate high-risk patients with poor overall survival from low-risk patients. According to three artificial intelligence algorithms, the current study developed an artificial intelligence survival predictive system that could provide three individual mortality risk curves for ovarian cancer.

**Conclusion:** In conclusion, the current study identified 1,307 differentially expressed genes and 337 differentially expressed immune genes in ovarian cancer patients. Multivariate Cox analyses identified fourteen prognostic immune biomarkers for ovarian cancer. The current study constructed an immune regulatory network involving 63 immune genes and 5 transcription factors, revealing potential regulatory associations among immune genes and transcription factors. The current study developed a prognostic model to predict the prognosis of ovarian cancer patients. The current study further developed two artificial intelligence predictive tools for ovarian cancer, which are available at https://zhangzhiqiao8.shinyapps.io/Smart_Cancer_Survival_Predictive_System_17_OC_F1001/ and https://zhangzhiqiao8.shinyapps.io/Gene_Survival_Subgroup_Analysis_17_OC_F1001/. An artificial intelligence survival predictive system could help improve individualised treatment decision-making.

## Introduction

Ovarian cancer (OC) is one of the most lethal malignant tumours in women, with 295,414 new cases and 184,799 deaths in 2018 (1). Although considerable progress has been made in diagnostic and therapeutic techniques, the 5-year survival rate of advanced OC patients remains poor (2). Early identification of patients with high mortality risk and more precise, individualised treatments will help improve the prognosis of OC patients. Regarding precision medicine, developing predictive models to provide early individualised mortality risk prediction and predicting the effectiveness of specific therapeutic schedules would be significant.

Considerable progress in bioinformatics helps scientists explore the intrinsic regulatory mechanisms of tumorigenesis and progression (3–6). The immune microenvironment plays an important role in the initiation and development of tumours (7, 8). Various studies have reported the clinical value of immunotherapy for ovarian cancer (5, 6). Several studies established prognostic models to predict the prognosis of OC patients (7, 8). However, regarding precision medicine, mortality risk prediction for high-risk and low-risk subgroups could not meet the needs of individualised treatment. Individualised treatment needs precise prognostic models to provide individual mortality risk prediction for a specific agent but not for a special subgroup.

Our team constructed two precision medicine predictive tools that predict individualised mortality risk for hepatocellular carcinoma (9, 10). These two precision medicine predictive tools provide online mortality risk prediction that is convenient and easy to understand. More importantly, these precision medicine predictive tools provide individual and specific mortality risk prediction, which is important for individualised treatment decision-making. Recently, artificial intelligence based on big data and advanced algorithms has been used to improve the accuracy of predictive models for the diagnosis and prognosis in various tumours (11–13). Therefore, the current study aimed to build artificial intelligence predictive tools to predict individualised mortality risk for OC patients based on immune genes.

## Materials and Methods

### Study Datasets

We retrieved the Gene Expression Omnibus (GEO) database according to the following conditions to obtain valuable research datasets: (1) The dataset should have available gene expression profile data; (2) The dataset should have complete clinicopathological data; (3) The dataset should have follow-up survival information. The GSE32062 dataset contained expression profiling data from 260 advanced-stage high-grade OC patients (https://www.ncbi.nlm.nih.gov/geo/query/acc.cgi?acc=GSE32062). The GSE53963 dataset contained expression profiling data from 174 high-grade OC patients (https://www.ncbi.nlm.nih.gov/geo/query/acc.cgi?acc=GSE53963). To eliminate the effect of death caused by non-tumour factors on the results of survival analysis, surviving patients with a survival time of <3 months were removed from the current study. Therefore, the GSE32062 dataset and GSE53963 dataset involved 420 patients, and 19,569 mRNAs were downloaded as model datasets for further survival. Probe IDs generated on the GPL6480 platform were converted to gene symbols based on Gencode v29. The TCGA cohort contained 21,586 mRNAs and 370 OC patients as a validation dataset for survival. The gene count values were log2-transformed for the TCGA cohort. The flow chart of patient selection is shown in Supplementary Figure 1.

### Differential Expression Analyses

We searched the GEO database to explore a dataset containing gene expression information of ovarian cancer samples and normal samples. The GSE26712 dataset was generated on the Affymetrix Human Genome U133A Array platform. The GSE26712 dataset has gene expression profiling information from 185 primary ovarian tumours and 10 normal ovarian surface epithelium (https://www.ncbi.nlm.nih.gov/geo/query/acc.cgi?acc=GSE26712). Therefore, differential expression analyses were performed between 185 tumour samples and 10 normal samples (GSE26712). Cut-off values for differential expression analyses were log_2_ |fold change| > 1 and *P* < 0.05. The data were normalised using the trimmed mean of M values method with “edgeR” (14).

### Immune Genes

The Immunology Database and Analysis Portal database were used to identify the immune gene list (15). Transcription factors were identified via the Cistrome Cancer database (16). Cytoscape v3.6.1 was used to develop an immune regulatory network of prognostic immune genes and their highly related transcription factors (11). Thresholds of |correlation coefficient| > 0.5 and *P* < 0.01 were used to identify transcription factors highly correlated with prognostic immune genes. The biological processes of immune genes were identified using the TISIDB database (http://cis.hku.hk/TISIDB/index.php).

### Tumour Immune Infiltration

Associations among tumour infiltrating immune cells and immune genes were evaluated by the Tumour Immune Estimation Resource database (16). Twenty-eight tumour immune infiltration scores were generated by single sample gene set enrichment analysis (17, 18).

### Statistical Analyses

Statistical analyses were conducted by SPSS Statistics 19.0 (SPSS Inc., USA). Artificial intelligence and bioinformatics analyses were performed using Python language 3.7.2 and R software 3.5.2 with the following artificial intelligence algorithms: random survival forest (RFS) algorithm (19, 20), multitask logistic regression (MTLR) algorithm (21, 22), and Cox survival regression algorithm (23, 24). The important packages included pec, rms, survival, rmda, ggplot2, GOplot, timereg, randomForestSRC, and riskRegression. The threshold for a statistically significant difference was a *P* < 0.05.

## Results

### Study Datasets

The clinical information of the OC patients is shown in Table 1. There were 229 (61.9%) of 370 patients who died in the TCGA cohort (validation dataset), and 260 (61.9%) of 420 patients died in the GEO cohort (model dataset). As shown in Table 1, there was no significant difference regarding mortality, survival time of deceased patients, or grade between the modelling cohort and the validation cohort during the follow-up period (*P* > 0.05). The overall survival time of all patients and the survival time of the patients in the survival subgroup for the model dataset were significantly longer than those of the validation dataset. However, the survival time of patients in the death subgroup of the model dataset was shorter than that of the patients in the death subgroup of the validated dataset, indicating that the difference in survival time between the two datasets might be related to the longer follow-up time of patients in the GEO cohort (model dataset).

**Table 1:** Clinical features of included patients.

	**TCGA dataset**	**GEO dataset**	***P*-value**
Number [*n*]	370	420	
Deaths [*n* (%)]	229 (61.9)	260 (61.9)	0.997
Total survival time (mean ± SD, month)	39.2 ± 31.0	47.9 ± 37.3	<0.001
Survival time for dead patients (month)	38.7 ± 25.7	36.8 ± 28.3	0.430
Survival time for living patients (month)	40.0 ± 38.1	66.0 ± 42.7	<0.001
Age (mean ± SD, year)	60.0 ± 11.0	NA	
Grade 4 [*n* (%)]	1 (0.3)	74 (17.6)	0.615
Grade 3 [*n* (%)]	316 (85.4)	212 (50.5)	
Grade 2 [*n* (%)]	42 (11.4)	134 (31.9)	
Grade 1 [*n* (%)]	1 (0.3)	0	
Grade (NA) [*n* (%)]	10 (2.7)	0	
Stage 4 [*n* (%)]	57 (15.4)	93 (22.1)	<0.001
Stage 3 [*n* (%)]	289 (78.1)	320 (76.2)	
Stage 2 [*n* (%)]	20 (5.4)	7 (1.7)	
Stage 1 [*n* (%)]	1 (0.3)	0	
Stage (NA) [*n* (%)]	3 (0.8)	0	
Vascular invasion (positive) [*n* (%)]	63 (17.0)	NA	
Vascular invasion (negative) [*n* (%)]	39 (10.5)	NA	
Vascular invasion (NA) [*n* (%)]	268 (72.4)	NA	
Lymphovascular invasion (positive) [*n* (%)]	99 (26.8)	NA	
Lymphovascular invasion (negative) [*n* (%)]	46 (12.4)	NA	
Lymphovascular invasion (NA) [*n* (%)]	225 (60.8)	NA	
ECOG score (2–4) [*n* (%)]	6 (1.6)	NA	
ECOG score (1) [*n* (%)]	26 (7.0)	NA	
ECOG score (0) [*n* (%)]	53 (14.3)	NA	
ECOG score (NA) [*n* (%)]	285 (77.0)	NA	

*Continuous variables were presented as mean ± standard deviation; NA, missing data; AJCC, American Joint Committee on Cancer*.

### Differential Expression Analyses

**Figure 1:**
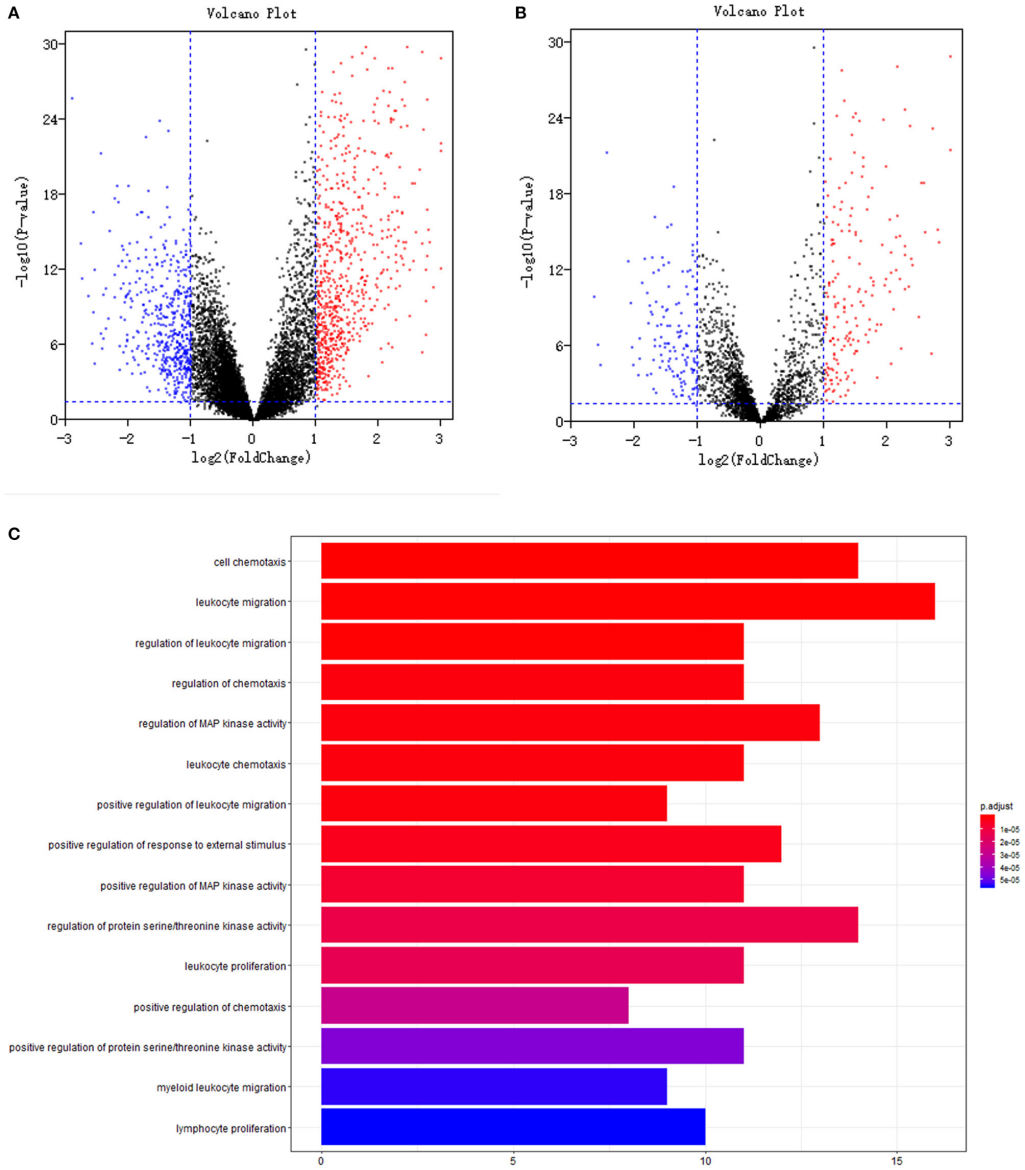
Differentially expression and functional enrichment: **(A)**. Volcano plot of all genes; **(B)**. Volcano plot of immune genes; **(C)**. Barplot chart of immune genes. The depth of the color represents different *P-*values; The length of the band represents the number of enriched genes.

Volcano plots of 13,216 mRNAs and 3,075 immune genes are shown in Figures 1A,B. With a threshold of log2 |fold change| > 1 and *P* < 0.05, differential expression analysis identified 779 upregulated and 528 downregulated mRNAs from 13,216 mRNAs (Figure 1A) between 185 tumour samples and 10 normal samples (GSE26712 dataset). Differential expression analysis further identified 194 upregulated and 143 downregulated immune mRNAs from 3,075 immune mRNAs (Figure 1B) between 185 tumour samples and 10 normal samples in the GSE26712 dataset.

To explore the gene expression difference of the identified immune biomarkers between the patients who died among the remaining patients with respect to the year of death, we further performed differential expression analysis between 130 tumour samples of patients who died and 10 normal samples of living patients (GSE26712 dataset). Differential expression analysis identified 753 upregulated and 526 downregulated mRNAs from 13,216 mRNAs. Differential expression analysis further identified 190 upregulated and 137 downregulated immune mRNAs from 3,075 immune mRNAs in the GSE26712 dataset.

### Functional Enrichment Analyses

**Figure 2:**
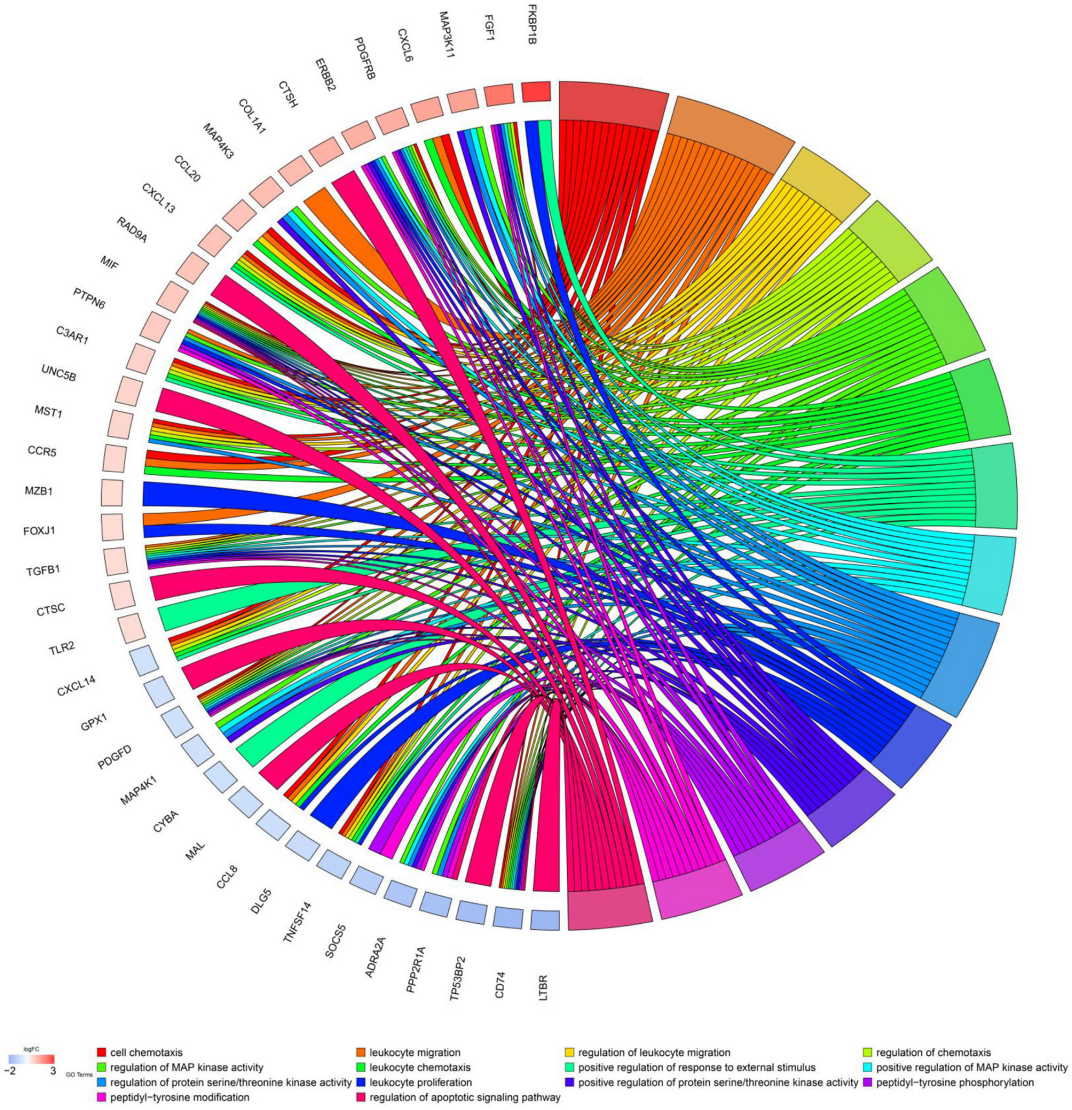
Chord chart of prognostic genes. Biological processes of previous 84 prognostic immune genes were mainly enriched in cell chemotaxis, leukocyte migration, regulation of protein serine/threonine kinase activity, regulation of MAP kinase activity, positive regulation of response to external stimulus, regulation of leukocyte migration, regulation of chemotaxis, leukocyte chemotaxis, positive regulation of MAP kinase activity, and leukocyte proliferation.

Further univariate Cox regression identified 84 prognostic immune gene biomarkers for OC patients in the model dataset (GSE32062 dataset and GSE53963 dataset). The bar plot (Figure 1C) and Gene Ontology chord chart (Figure 2) showed that the biological processes of the previous 84 prognostic immune genes were mainly enriched in leukocyte migration, cell chemotaxis, regulation of protein serine/threonine kinase activity, regulation of MAP kinase activity, positive regulation of response to external stimulus, regulation of leukocyte migration, regulation of chemotaxis, leukocyte chemotaxis, positive regulation of MAP kinase activity, and leukocyte proliferation. The results of the bar plot and Gene Ontology chord chart suggested that the above biological processes might play a role in the occurrence, growth, invasion, and prognosis of ovarian cancer, and the underlying mechanism is worthy of further study.

### Immune Regulatory Network

**Figure 3:**
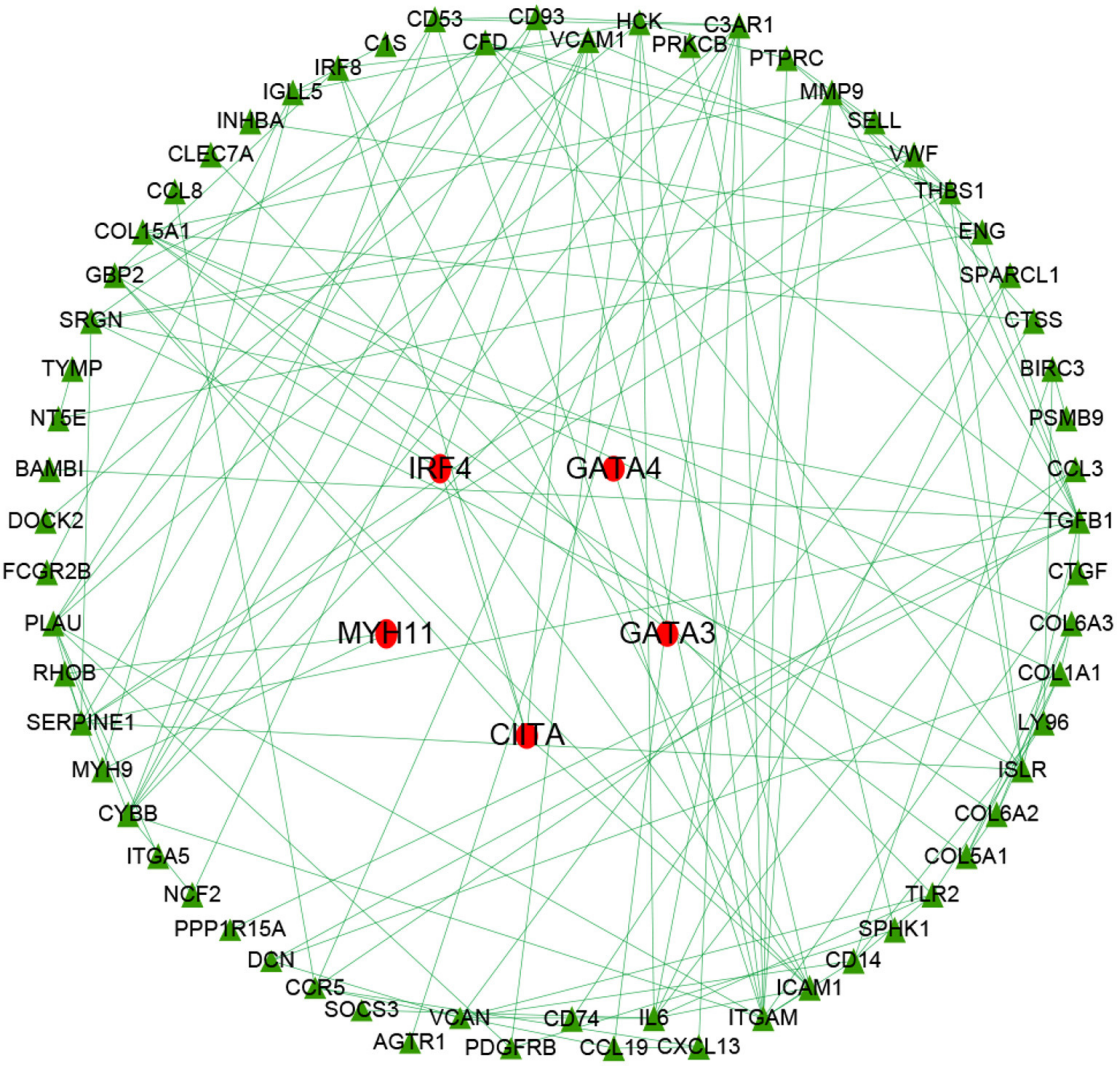
Immune gene regulatory network chart. The immune regulatory network involved 63 immune genes and 5 transcription factors. IRF4, GATA4, GATA3, CIITA, and MYH11 were involved in the immune regulatory network, indicating these transcription factors might play a role in the immune microenvironment of ovarian cancer.

Univariate Cox regression identified 84 prognostic immune biomarkers for the OS of OC patients. Transcription factors that were highly correlated with prognostic immune mRNAs were identified with previous correlation analysis thresholds. To explore the potential regulatory relationships among these immune genes, these previous prognostic immune mRNAs and their highly correlated transcription factors were placed in the STRING database with confidence values of 0.90. Thus, a regulatory network involving 63 immune genes and 5 transcription factors was constructed by using Cytoscape v3.6.1 (Figure 3). As shown in Figure 3, IRF4, GATA4, GATA3, CIITA, and MYH11 were involved in the immune regulatory network, indicating that these five transcription factors might play a role in the immune microenvironment of ovarian cancer.

### Construction of a Prognostic Model

**Figure 4:**
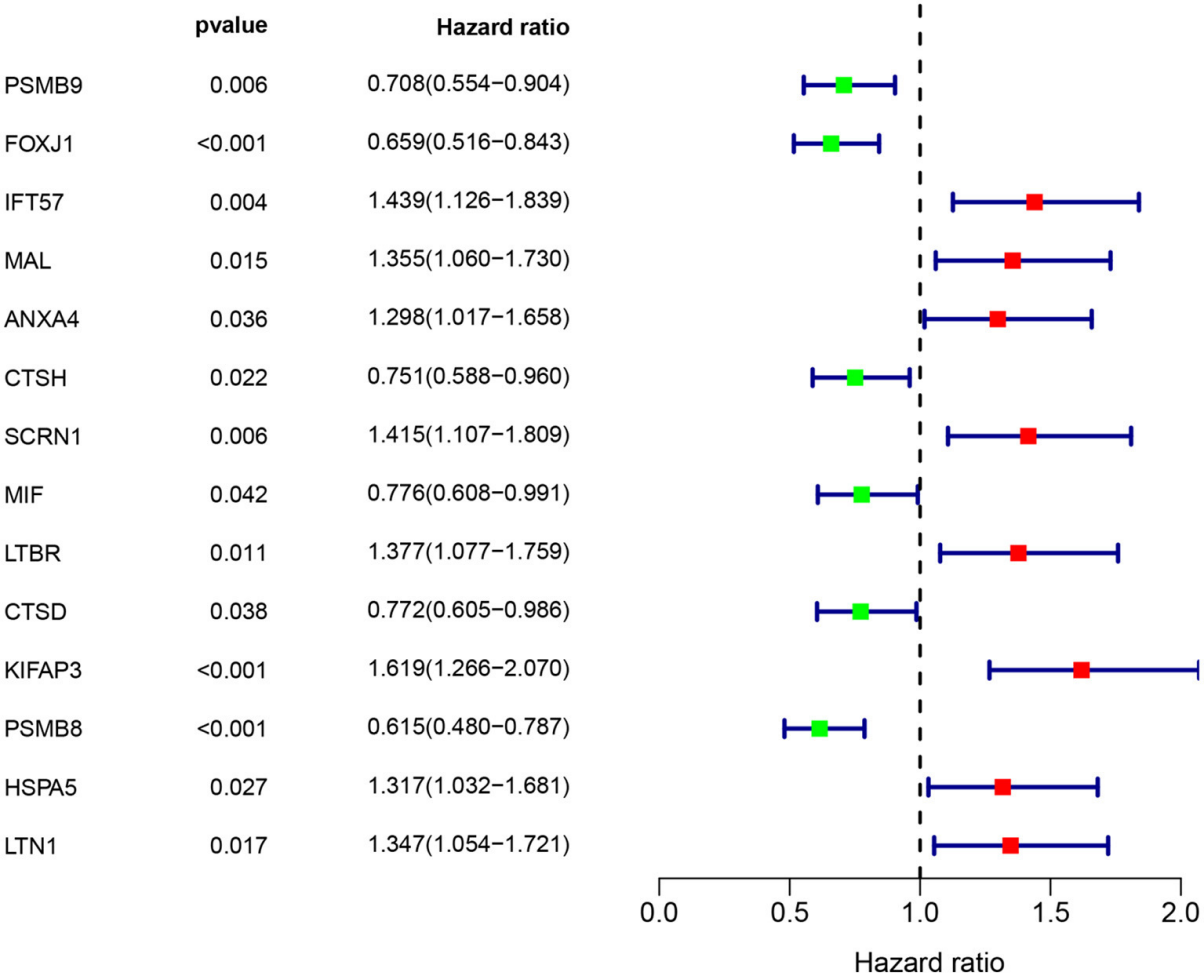
Immune gene survival forest chart. Eight immune factors (IFT57, MAL, ANXA4, SCRN1, LTBR, KIFAP3, HSPA5, and LTN1) were positively correlated with poor prognosis of ovarian cancer, whereas six immune factors (PSMB9, FOXJ1, CTSH, MIF, CTSD, and PSMB8) were negatively correlated with poor prognosis of ovarian cancer.

**Figure 5:**
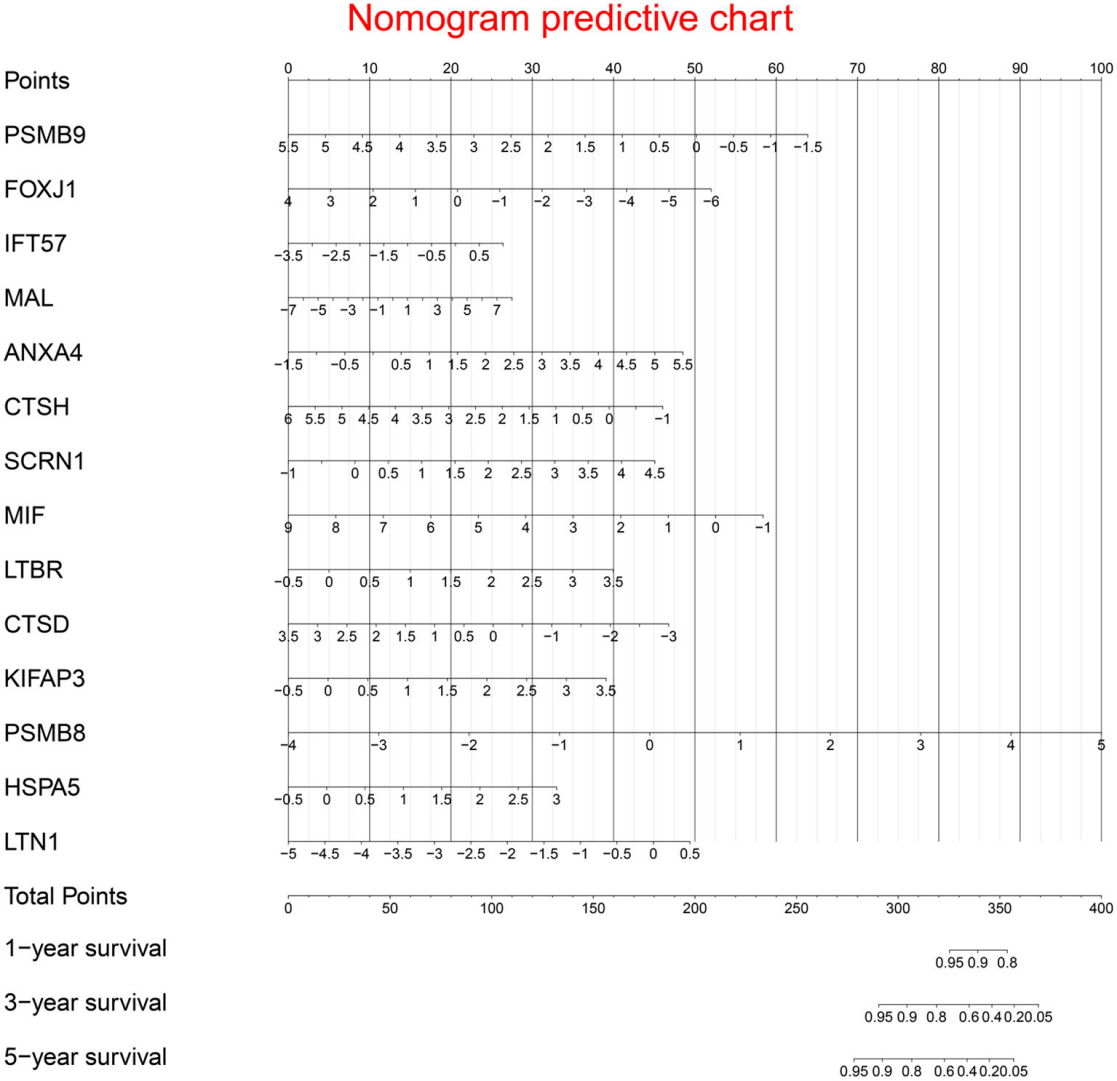
Prognostic nomogram chart. For each prognostic gene, different gene expression values were assigned different risk scores. The total point (overall risk score) of one patient was obtained by adding up the risk scores of 14 prognostic genes. Through the vertical line corresponding to the total point, we can obtain the corresponding mortality rate of individual patient at different times.

Multivariate Cox regression identified fourteen independent prognostic mRNAs for OS (Table 2 and Figure 4), indicating that these 14 prognostic immune genes might be more closely related to the prognosis of ovarian cancer than the prognostic immune genes that were not included in multivariate Cox regression. The formula of the prognostic model based on multivariate Cox regression was as follows: prognostic score = (-0.472^*^PSMB9) + (-0.268^*^FOXJ1) + (0.303^*^IFT57) + (0.095^*^MAL) + (0.357^*^ANXA4) + (-0.339^*^CTSH) + (0.422^*^SCRN1) + (-0.301^*^MIF) + (0.515^*^LTBR) + (-0.371^*^CTSD) + (0.503^*^KIFAP3) + (0.574^*^PSMB8) + (0.485^*^HSPA5) + (0.463^*^LTN1). A prognostic nomogram is shown in Figure 5. For each prognostic gene, different gene expression values were assigned different risk scores. The total points (overall risk score) of one patient were obtained by adding up the risk scores of 14 prognostic genes. Through the vertical line corresponding to the total points, we can obtain the corresponding mortality rate of individual patients at different times.

**Table 2:** Information of prognostic immune genes.

	**Univariate analysis**			**Multivariate analysis**			
**Immune gene**	**HR**	**95% CI**	***P*-value**	**Coefficient**	**HR**	**95% CI**	***P*-value**
PSMB9 (High/Low)	0.708	0.554–0.904	0.006	−0.472	0.624	0.449–0.867	0.005
FOXJ1 (High/Low)	0.659	0.516–0.843	<0.001	−0.268	0.765	0.672–0.871	<0.001
IFT57 (High/Low)	1.439	1.126–1.839	0.004	0.303	1.354	1.014–1.807	0.040
MAL (High/Low)	1.355	1.060–1.730	0.015	0.095	1.099	1.019–1.186	0.014
ANXA4 (High/Low)	1.298	1.017–1.658	0.036	0.357	1.430	1.123–1.820	0.004
CTSH (High/Low)	0.751	0.588–0.960	0.022	−0.339	0.712	0.565–0.898	0.004
SCRN1 (High/Low)	1.415	1.107–1.809	0.006	0.422	1.525	1.126–2.065	0.006
MIF (High/Low)	0.776	0.608–0.991	0.042	−0.301	0.740	0.602–0.910	0.004
LTBR (High/Low)	1.377	1.077–1.759	0.011	0.515	1.674	1.190–2.354	0.003
CTSD (High/Low)	0.772	0.605–0.986	0.038	−0.371	0.690	0.523–0.912	0.009
KIFAP3 (High/Low)	1.619	1.266–2.070	<0.001	0.503	1.653	1.189–2.298	0.003
PSMB8 (High/Low)	0.615	0.480–0.787	<0.001	0.574	1.775	1.165–2.703	0.008
HSPA5 (High/Low)	1.317	1.032–1.681	0.027	0.485	1.625	1.094–2.415	0.016
LTN1 (High/Low)	1.347	1.054–1.721	0.017	0.463	1.588	1.051–2.400	0.028

*HR, hazard ratio; CI, confidence interval. The medians of gene expression values were used as cut-off values to stratify gene expression values into high expression group (as value 1) and low expression group (as value 0)*.

Supplementary Figure 2 shows significant differences in survival curves between the high-risk group and the low-risk group. Eight immune factors (IFT57, MAL, ANXA4, SCRN1, LTBR, KIFAP3, HSPA5, and LTN1) were positively correlated with poor prognosis of ovarian cancer, whereas six immune factors (PSMB9, FOXJ1, CTSH, MIF, CTSD, and PSMB8) were negatively correlated with poor prognosis of ovarian cancer. Supplementary Figures 3, 4 show the predictive value distribution chart and the survival status scatter plot.

### Performance of Model Cohort

**Figure 6:**
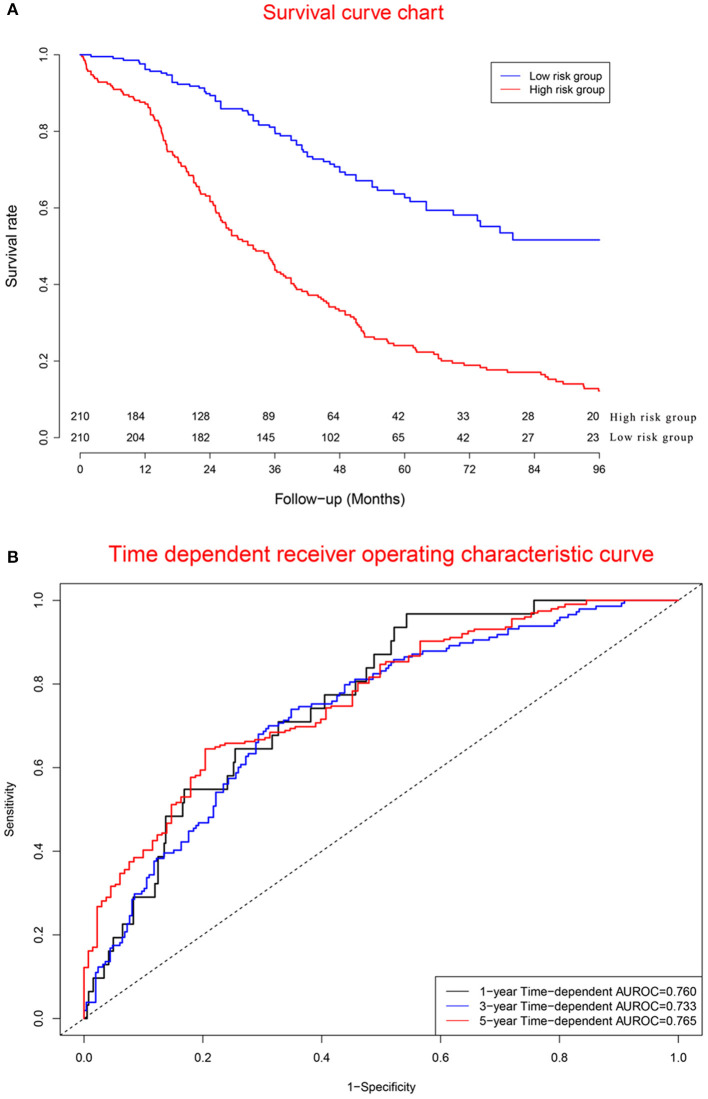
Clinical performance in model cohort: **(A)**. Survival curves for high risk group and low risk group; **(B)**. Time-dependent receiver operating characteristic curves. The mortality rate in the high risk group was significantly higher than that in the low risk group. Concordance indexes were 0.760, 0.733, and 0.765 for 1-, 3-, and 5-year survival, indicating that the prognostic model has a good predictive value for the prognosis of ovarian cancer patients.

Survival curves of the two groups are illustrated in Figure 6A, showing that the mortality rate in the high-risk group was significantly higher than that in the low-risk group. Concordance indexes were 0.760, 0.733, and 0.765 for 1-, 3-, and 5-year survival, respectively (Figure 6B), indicating that the prognostic model has good predictive value for the prognosis of OC patients. Supplementary Figure 5 shows the calibration curves of the model cohort, showing that there was good consistency between the predicted mortality rate and the actual mortality rate.

### Performance of Validation Cohort

**Figure 7:**
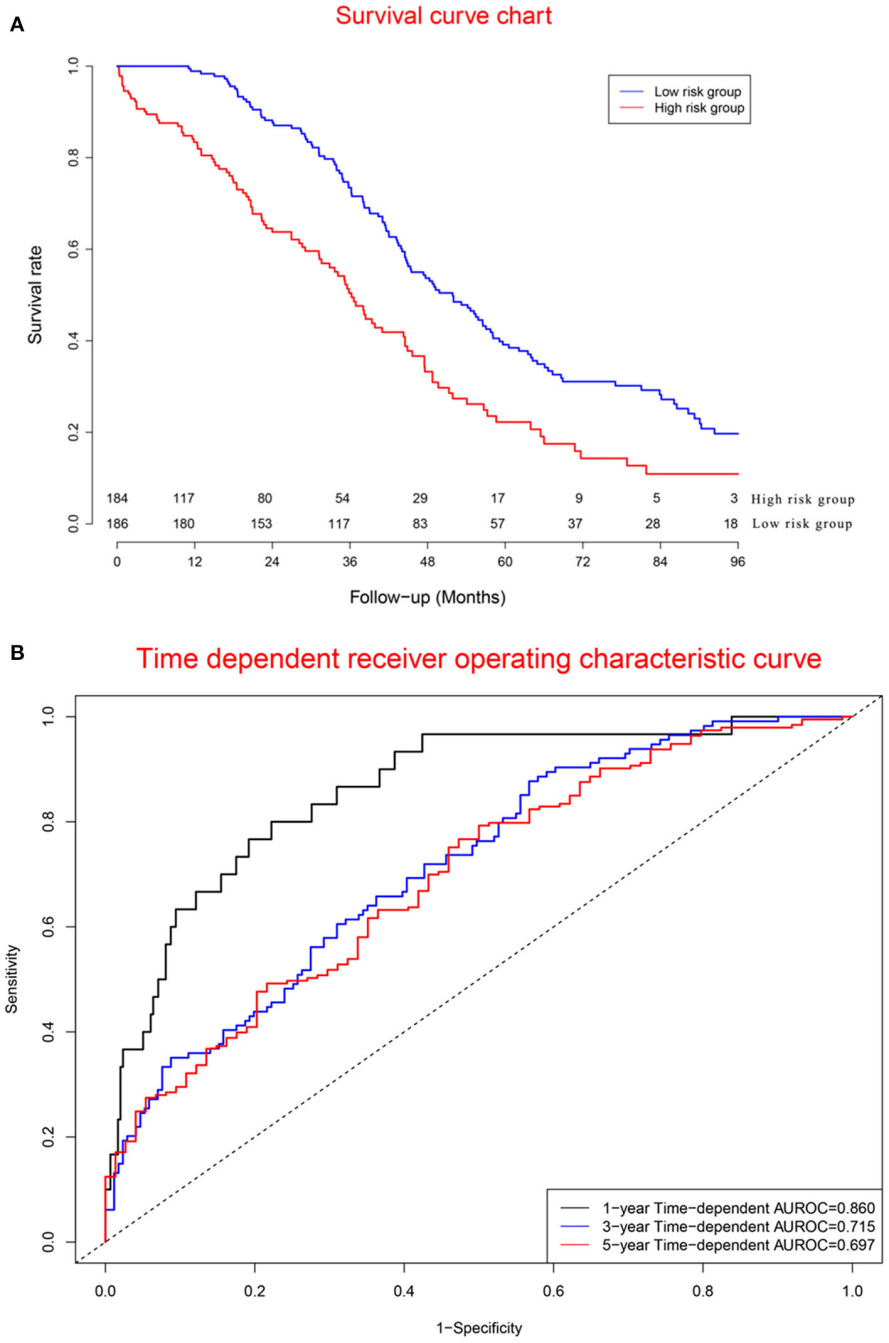
Clinical performance in validation cohort: **(A)**. Survival curves for high risk group and low risk group; **(B)**. Time-dependent receiver operating characteristic curves. The mortality rate in the high risk group was significantly higher than that in the low risk group. Concordance indexes were 0.860, 0.715, and 0.679 for 1-, 3-, and 5-year survival, respectively **(B)**, indicating that the prognostic model has a good predictive value for the prognosis of ovarian cancer patients.

Survival curves of the two groups are illustrated in Figure 7A, showing that the mortality rate in the high-risk group was significantly higher than that in the low-risk group. Concordance indexes were 0.860, 0.715, and 0.679 for 1-, 3-, and 5-year survival, respectively (Figure 7B), indicating that the prognostic model has good predictive value for the prognosis of OC patients. Supplementary Figure 6 shows calibration curves of the validation cohort. Supplementary Figure 7 shows decision curves for 1-, 3-, and 5-year survival, showing that there was consistency between the predicted mortality rate and the actual mortality rate.

### Artificial Intelligence Survival Predictive System

**Figure 8:**
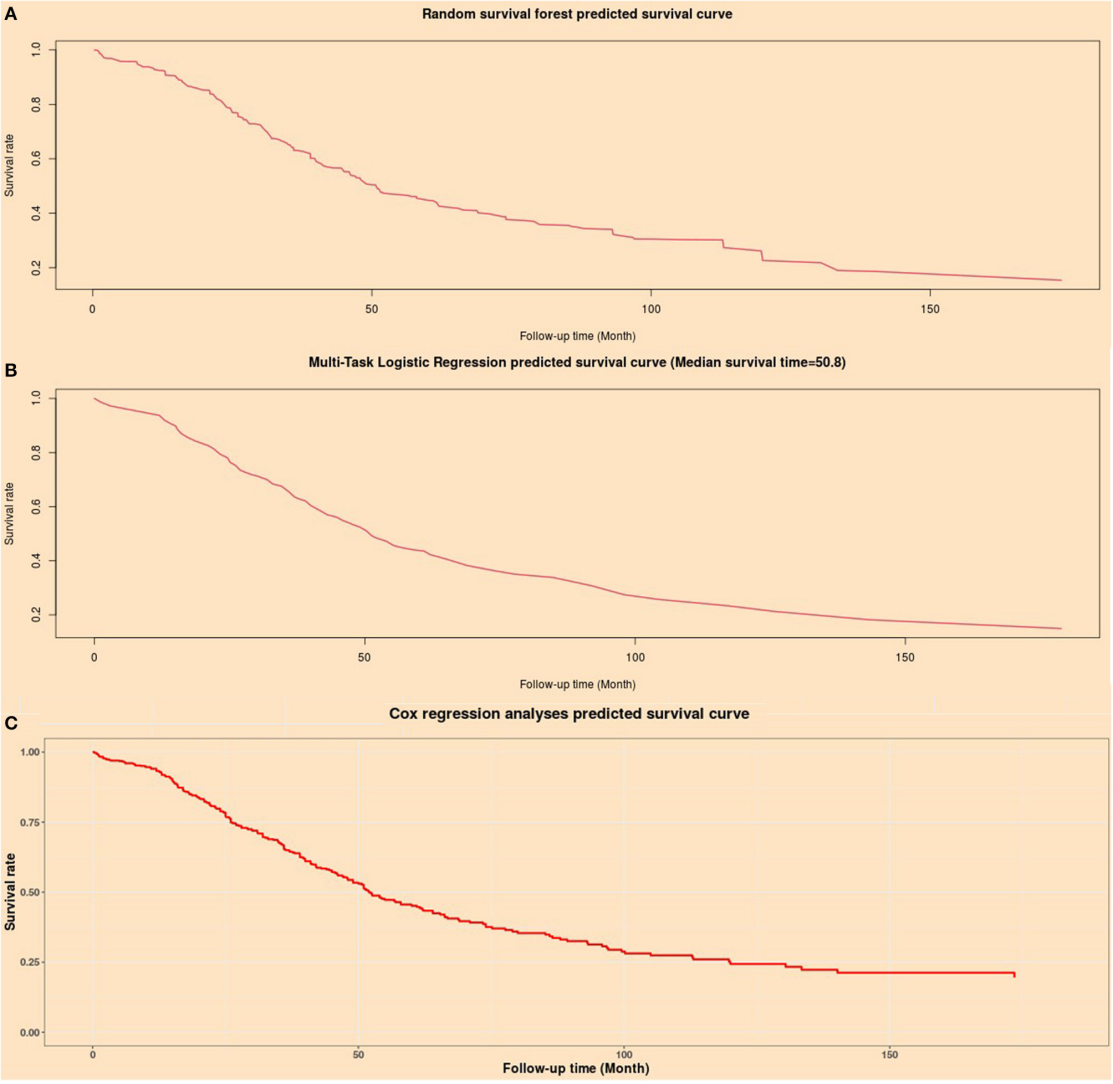
Individual mortality risk predictive curves based on artificial intelligence algorithms. **(A)** Random survival forest model; **(B)** Multitask logistic regression model; **(C)** Cox proportional hazard regression model.

An artificial intelligence survival prediction system was constructed for individual mortality risk prediction for OC patients (Figure 8) and is available at https://zhangzhiqiao8.shinyapps.io/Smart_Cancer_Survival_Predictive_System_17_OC_F1001/. After the user inputs the expression values of the prognostic genes and clicks the “predict” button, the survival curve of one individual patient during the follow-up period will be presented.

The artificial intelligence survival prediction system provides three individual mortality risk predictive curves based on artificial intelligence algorithms: the RFS model (Figure 8A), MTLR model (Figure 8B), and Cox model (Figure 8C).

### Gene Survival Analysis Screen System

A Gene Survival Analysis Screen System was constructed for exploratory research of immune genes (Supplementary Figure 8) and is available at https://zhangzhiqiao8.shinyapps.io/Gene_Survival_Subgroup_Analysis_17_OC_F1001/. After the user inputs the parameters and clicks the “survival curve analysis” button, the survival curves of the high-risk group and low-risk group are presented. Users can obtain hazard ratio values of different clinical parameters after clicking the “Univariate Cox survival analysis table” button in the Gene Survival Analysis Screen System.

### Independence Assessment

We used multivariate Cox regression to explore the independent effect of the prognostic model on the prognosis of OC patients. The prognostic signature was an independent influencing factor for OS in the model cohort (Table 3). In the validation cohort, the prognostic signature was an independent risk factor for OS. The results of multivariate Cox regression showed that the prognostic model had an independent effect on the prognosis of ovarian cancer, which further supported the value of the prognostic model in predicting ovarian cancer prognosis.

**Table 3:** Results of Cox regression analyses.

	**Univariate analysis**			**Multivariate analysis**			
	**HR**	**95% CI**	***P*-value**	**Coefficient**	**HR**	**95% CI**	***P*-value**
**Model cohort (*****n*** **=** **420)**							
AJCC Stage (3–4/1–2)	1.913	0.783–4.679	0.155	1.000	2.718	1.111–6.646	0.028
AJCC Grade (3–4/1–2)	1.417	1.066–1.882	0.016	0.108	1.114	0.832–1.492	0.468
Prognostic model (High/Low)	2.988	2.294–3.893	<0.001	1.105	3.019	2.302–3.959	<0.001
**Validation cohort (*****n*** **=** **370)**							
AJCC Stage (3–4/1–2)	1.999	0.887–4.505	0.095	0.734	2.084	0.913–4.756	0.081
AJCC Grade (3–4/1–2)	1.209	0.808–1.812	0.356	0.046	1.048	0.695–1.579	0.824
Prognostic model (High/Low)	1.915	1.466–2.500	<0.001	0.658	1.930	1.472–2.531	<0.001

*AJCC, the American Joint Committee on Cancer; HR, hazard ratio; CI, confidence interval. The median of Prognostic model scores was used as the cut-off value to stratify gastric cancer patients into high risk group and low risk group*.

## Discussion

The current study identified 1,307 differentially expressed genes and 337 differentially expressed immune genes between tumour samples and normal samples. Further univariate Cox regression identified 84 prognostic immune gene biomarkers for OC patients in the model dataset (GSE32062 dataset and GSE53963 dataset). An immune regulatory network was depicted involving 63 immune genes and 5 transcription factors. Through bioinformatics research, the current study depicted potential regulatory relationships among immune genes and transcription factors. Fourteen immune genes were identified as independent prognostic factors by multivariate survival analysis. Kaplan-Meier survival curves showed that these 14 prognostic genes were closely related to the prognosis of ovarian cancer patients. These 14 prognostic genes were used to develop a prognostic nomogram for ovarian cancer. Moreover, two artificial intelligence predictive tools were developed for precise individual mortality risk prediction in ovarian cancer. Based on a random survival forest algorithm, a multitask logistic regression algorithm, and a Cox survival regression algorithm, the current artificial intelligence survival predictive system provided three individual mortality risk predictive curves for the evaluation and improvement of individualised medical decisions.

In the current study, 1,308 differentially expressed genes (including 337 differential immune genes) were identified by differential expression analysis. Compared with normal ovarian tissues, these differentially expressed genes showed high expression or low expression in tumour tissues, suggesting that these differentially expressed genes might be related to the biological characteristics and clinical process of OC. Further univariate Cox and multivariate Cox regression analyses identified 84 and 14 prognostic immune genes, respectively, suggesting that these 14 prognostic immune genes might be closely related to the prognosis of OC patients. Functional enrichment analysis showed that the 84 genes were mainly related to the regulation of immune inflammation and were enriched in leukocyte migration, cell chemotaxis, regulation of protein serine/threonine kinase activity, and regulation of MAP kinase activity.

The immune regulatory network further indicated the potential regulatory relationship among 63 immune genes and 5 transcription factors, suggesting that these immune genes and transcription factors might play a potential role in the regulatory mechanism of the tumour immune environment. Previous studies have provided supporting evidence for the potential mechanisms of these five transcription factors regarding tumour growth, progression and prognosis. There is a close relationship between GATA3 and poor prognosis of high-grade serous ovarian carcinoma (25). GATA3 positivity is associated with poor prognosis of pancreatic ductal adenocarcinoma (26). High expression of GATA3 is associated with good prognosis of ER+ breast cancer (27). IRF4 might activate the Notch-Akt signalling pathway in non-small cell lung cancer (28). Higher expression of IRF4+ Tregs was related to poor prognosis for different cancers (29). IRF4 was an independent prognostic factor for node-negative breast cancer (30). MYH11 positively modulated the immune-related gene GLP2R in colon adenocarcinoma (31). MYH11 positively regulated GSTM5, PTGIS, ENPP2, and P4HA3 (32). GATA4 inhibits tumour growth by affecting the assembly of tumour suppressor enhancement modules (33). Overexpression of GATA4 can protect human granulosa cell tumours from apoptosis induced by TRAIL *in vitro* (34).

Different research teams have established valuable survival prediction models for ovarian cancer based on different research cohorts and modelling methods. Previous prognostic models provided mortality curves for two classes of patients with different clinical characteristics (7, 8) but did not provide mortality curves for individual patients. He et al. constructed a prognostic model based on 10 RNA-binding proteins for ovarian cancer (35). However, the calculation formula of this model is so complex that it is difficult for patients to calculate their personal risk score. Bing et al. constructed a novel model by merging three previous models selected by the integrated *P*-value method, providing a new idea for the establishment of a prognostic model (36). However, this theoretically feasible method has not been applied in clinical research because it involves the fusion of multiple prognostic models. Tang et al. presented an eight-mRNA prognostic model for ovarian cancer (37), providing a valuable predictive model for clinical practise. If the above models can provide a simple calculation tool, it will be more helpful to provide convenient survival prediction information for patients with ovarian cancer. In fact, every cancer patient cares only for her or his own individual mortality after diagnosis. Due to the considerable clinical heterogeneity of tumours, clinicians observe large differences in clinical prognosis among different cancer patients. Therefore, it is of great significance to predict the individual mortality risk of cancer patients. The emergence of big data and advanced algorithms has laid a solid foundation for artificial intelligence research. Different artificial intelligence algorithms have been used to improve clinical diagnosis and prognostic prediction (11–13). Based on the artificial intelligence algorithms provided in previous studies, the current study developed an artificial intelligence survival prediction system. The current artificial intelligence survival prediction system provides three individual mortality risk predictive curves according to different artificial intelligence algorithms. These artificial intelligence algorithms are not widely used in clinical research because of the complexity of calculation. To the best of our knowledge, our team is the first to introduce various artificial intelligence algorithms for tumour prognosis research. Our study showed that artificial intelligence algorithms have great application value and superiority in predicting the individual mortality risk for cancer patients and are worth further research and application. The tumour immune microenvironment is reportedly related to oncogenesis and prognosis (7, 38). The current study revealed the potential association of tumour-infiltrating immune cells and immune genes with tumour prognosis. Compared with several previous predictive models for the prognosis of OC patients (14, 39), our precision medical predictive tools were more valuable in providing individual mortality risk prediction at different time points.

The TISIDB database was used to explore the biological processes of immune genes. The top biological processes of proteasome subunit beta 9 (PSMB9) were immune response-activating signal transduction, the immune response-regulating signalling pathway, and the immune response-activating cell surface receptor signalling pathway. The top biological processes of Forkhead box J1 (FOXJ1) were adaptive immune responses, leucocyte-mediated immunity, humoural immune response mediated by circulating immunoglobulin, and lymphocyte-mediated immunity. The top biological processes of mal, T-cell differentiation protein (MAL) were the extrinsic apoptotic signalling pathway via death domain receptors, regulation of apoptotic signalling pathway, and the extrinsic apoptotic signalling pathway. The top biological processes of annexin A4 (ANXA4) were interleukin-8 production, regulation of interleukin-8 production, and negative regulation of interleukin-8 production. The top biological processes of cathepsin H (CTSH) were T cell-mediated immunity, lymphocyte-mediated immunity, leucocyte-mediated immunity, and adaptive immune response. The top biological processes of macrophage migration inhibitory factor (MIF) were negative regulation of immune system process, B cell homeostasis, regulation of immune effect or process, and lymphocyte homeostasis. The top biological processes of lymphotoxin beta receptor (LTBR) were myeloid dendritic cell activation, leucocyte differentiation, response to tumour necrosis factor, and response to molecules of bacterial origin. The top biological processes of cathepsin D (CTSD) were autophagy, antigen processing and presentation of exogenous antigen, antigen processing and presentation of exogenous peptide antigen via MHC class II. The top biological processes of kinesin-associated protein 3 (KIFAP3) were antigen processing and presentation, antigen processing and presentation of peptide antigen via MHC class II, and antigen processing and presentation of exogenous antigen. The top biological processes of proteasome subunit beta 8 (PSMB8) were immune response-activating signal transduction, innate immune response-activating signal transduction, and the immune response-regulating cell surface receptor signalling pathway.

PSMB9, FOXJ1, IFT57, MAL, ANXA4, CTSH, SCRN1, MIF, LTBR, CTSD, KIFAP3, PSMB8, HSPA5, and LTN1 were recognised as independent risk factors by multivariate Cox analyses, suggesting that these 14 prognostic immune genes might have potential effects on the occurrence, progression and prognosis of tumours. NANOG controls cell migration and invasion by regulating FOXJ1 expression in ovarian cancer (15). FOXJ1 promoted tumour growth in bladder cancer (16). Highly expressed FOXJ1 promoted the proliferation and invasiveness of laryngeal squamous cell carcinoma cells (17). High expression of MAL was associated with poor survival of advanced ovarian cancer (40). Overexpression of the MAL gene was used to predict chemoresistance and poor prognosis in serous ovarian cancer patients (18). High expression of MAL promoted metastasis in colorectal cancer (24). Ikaros inhibited the proliferation of tumour cells by downregulating the expression of ANXA4 in hepatocellular carcinoma (23). Knockdown of SCRN1 significantly reduced tumour cell growth in colorectal cancer (19). EIF expression was associated with overall survival in patients with ovarian cancer (20). The KIFAP3 gene is highly expressed at the mRNA and protein levels in breast cancer (41). miR-451a inhibited cancer growth and induced apoptosis of papillary thyroid cancer by targeting PSMB8 (41). The CpG mutation of PSMB9 is related to the recurrence or drug resistance of ovarian cancer after chemotherapy (42). High expression of PSMB8 and PSMB9 is related to the five-year survival of ovarian cancer (43). High expression of MIF is correlated with poor overall survival of ovarian cancer (44). HSPA5 inhibits the growth of epithelial ovarian cancer cells through G1 phase arrest (45). High expression of CD5L promoted proliferation and the antiapoptotic response in hepatocellular carcinoma cells by binding to HSPA5 (46).

CD4 T helper cells can inhibit the transformation of immunosuppressive regulatory T cells in ovarian cancer (41). Regulatory T cells were positively correlated with ovarian cancer (20). An increased CD8/regulatory T cell ratio suggests good prognosis for ovarian cancer (47). Dendritic cell immunotherapy could stimulate antitumour T cell immunity and improve the prognosis of cancer patients (21). Interleukin 10 regulates Toll-like receptor-mediated dendritic cell activation in ovarian cancer (22). IL-15 enhanced natural killer cell function in ovarian cancer patients (13). A low lymphocyte-to-monocyte ratio was related to poor survival in ovarian cancer (48). Mast cell infiltration with high mean vessel density indicated favourable prognosis in ovarian cancer (49). Macrophage secretory proteins induce ovarian cancer proliferation through the JAK2/STAT3 pathway (50). M1 macrophages induce ovarian cancer cell metastasis through the activation of NF-κB (51). Small extracellular vesicles could inhibit the T cell response and promote the growth of ovarian cancer cells (51). Artesunate induced apoptosis of ovarian cancer cells by microRNA-142 (52). Mature neutrophils inhibited T cell immunity in ovarian cancer patients (50). Regulatory T cells inhibit CD8 T cell function through the IL-10 pathway (53). ISG15 induced CD8 T cells and inhibited the progression of ovarian cancer (54). TGF-beta 1 induces CD8 Tregs through the p38 MAPK pathway in ovarian cancer (55). CD4 T helper cells inhibit the transformation of immunosuppressive regulatory T cells (56). CD4 T cells induce the host immune response through dendritic cells in patients with MHC class II-negative ovarian cancer (57).

Advantages: First, the current study developed two artificial intelligence predictive tools that provided individual mortality risk prediction at different time points and were valuable for optimising individual treatment decisions. Second, the current artificial intelligence survival predictive system provided three individual mortality risk predictive curves based on three artificial intelligence algorithms. Different artificial intelligence algorithms provided more reliable and valuable prognostic predictions for ovarian cancer than conventional prognostic models.

Shortcomings: First, because study datasets from public databases did not include information on surgical treatment, radiotherapy, biological targeting therapy, etc., the current study failed to assess the impact of these important clinical variables on survival. Second, from the perspective of model validity and extensibility, the sample size of the current research was relatively small for prognosis, which might weaken the validity of the research conclusions. Large, prospective sample studies can provide more convincing clinical evidence for the current study. Third, as non-parametric algorithms, artificial intelligence algorithms are complex to perform, and their calculation processes cannot be expressed by simple equations, restricting artificial intelligence algorithms as the mainstream methods for prognostic studies. Fourth, the current study constructed an immune regulatory network and revealed potential regulatory associations among immune genes and transcription factors. However, the role and mechanism of immune genes and transcription factors in tumorigenesis, growth and prognosis need to be elucidated by further study.

In conclusion, the current study identified 1,307 differentially expressed genes and 337 differentially expressed immune genes in ovarian cancer patients. Multivariate Cox analyses identified fourteen prognostic immune biomarkers for ovarian cancer. The current study constructed an immune regulatory network involving 63 immune genes and 5 transcription factors, revealing potential regulatory associations among immune genes and transcription factors. The current study developed a prognostic model to predict the prognosis of ovarian cancer patients. The current research further developed two artificial intelligence predictive tools for ovarian cancer, which are available at https://zhangzhiqiao8.shinyapps.io/Smart_Cancer_Survival_Predictive_System_17_OC_F1001/ and https://zhangzhiqiao8.shinyapps.io/Gene_Survival_Subgroup_Analysis_17_OC_F1001/. The artificial intelligence survival predictive system can improve individualised treatment decision-making.

## Data Availability Statement

The original contributions presented in the study are included in the article/Supplementary Material, further inquiries can be directed to the corresponding author/s.

## Ethics Statement

Ethical review and approval was not required for the study on human participants in accordance with the local legislation and institutional requirements. Written informed consent for participation was not required for this study in accordance with the national legislation and the institutional requirements.

## Author Contributions

ZZ, TH, PW, JL, and LH: conceptualisation, methodology, resources, investigation, data curation, formal analysis, validation, software, project administration, and supervision. ZZ and PW: writing and visualisation. ZZ: funding acquisition. All authors contributed to the article and approved the submitted version.

## Conflict of Interest

The authors declare that the research was conducted in the absence of any commercial or financial relationships that could be construed as a potential conflict of interest.

## Abbreviations

OC: ovarian cancer
TCGA: The Cancer Genome Atlas
GEO: Gene Expression Omnibus
ROC: receiver operating characteristic
DFS: disease-free survival
HR: hazard ratio
CI: confidence interval
AJCC: American Joint Committee on Cancer
SD: standard deviation.
