# Supplementary material for: Potential Prognostic Immune Biomarkers of Overall Survival in Ovarian Cancer Through Comprehensive Bioinformatics Analysis: A Novel Artificial Intelligence Survival Prediction System
Source: Front Med (Lausanne). 2021 May 24;8:587496. doi: 10.3389/fmed.2021.587496 (PMC8180546; doi:10.3389/fmed.2021.587496)
Supplement: Supplementary file 1 [file Data_Sheet_1.PDF]

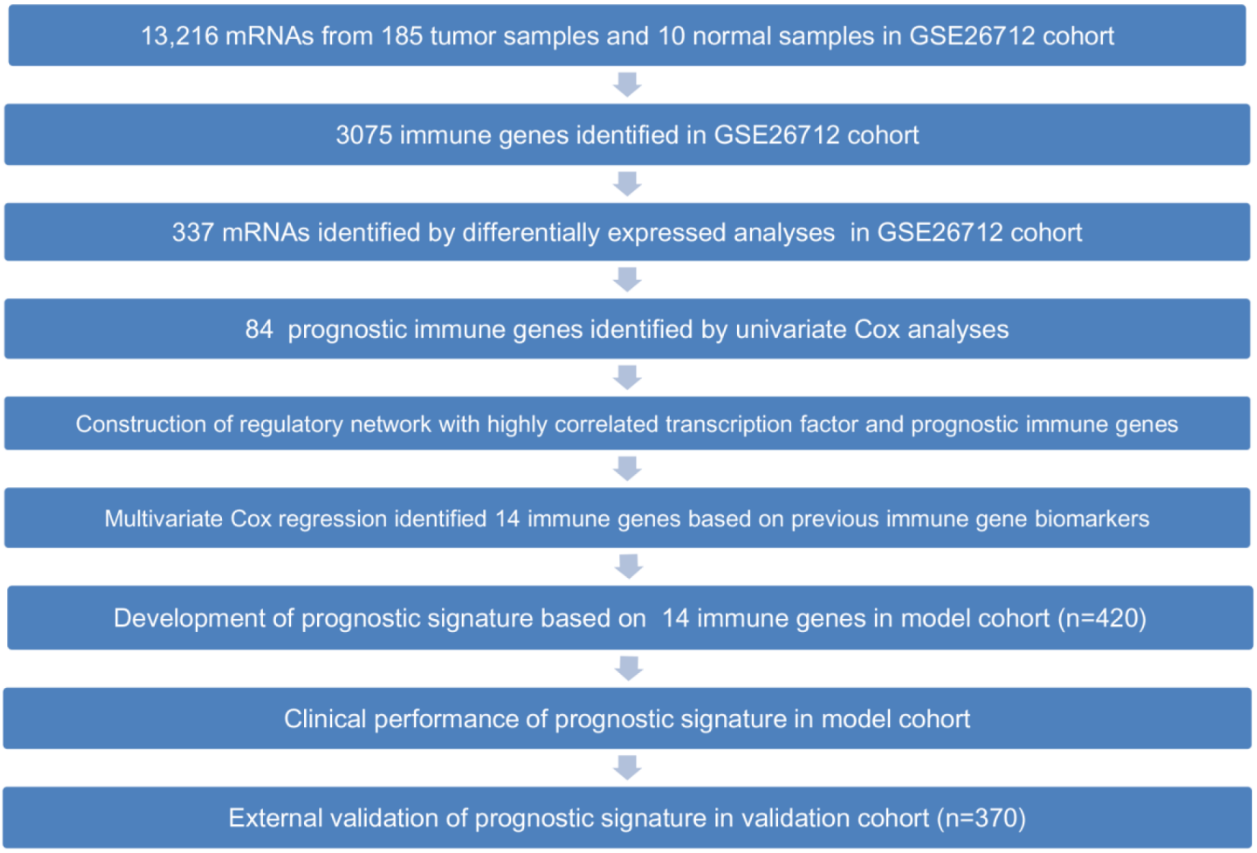

Supplementary Figure 1. Flow chart in current study

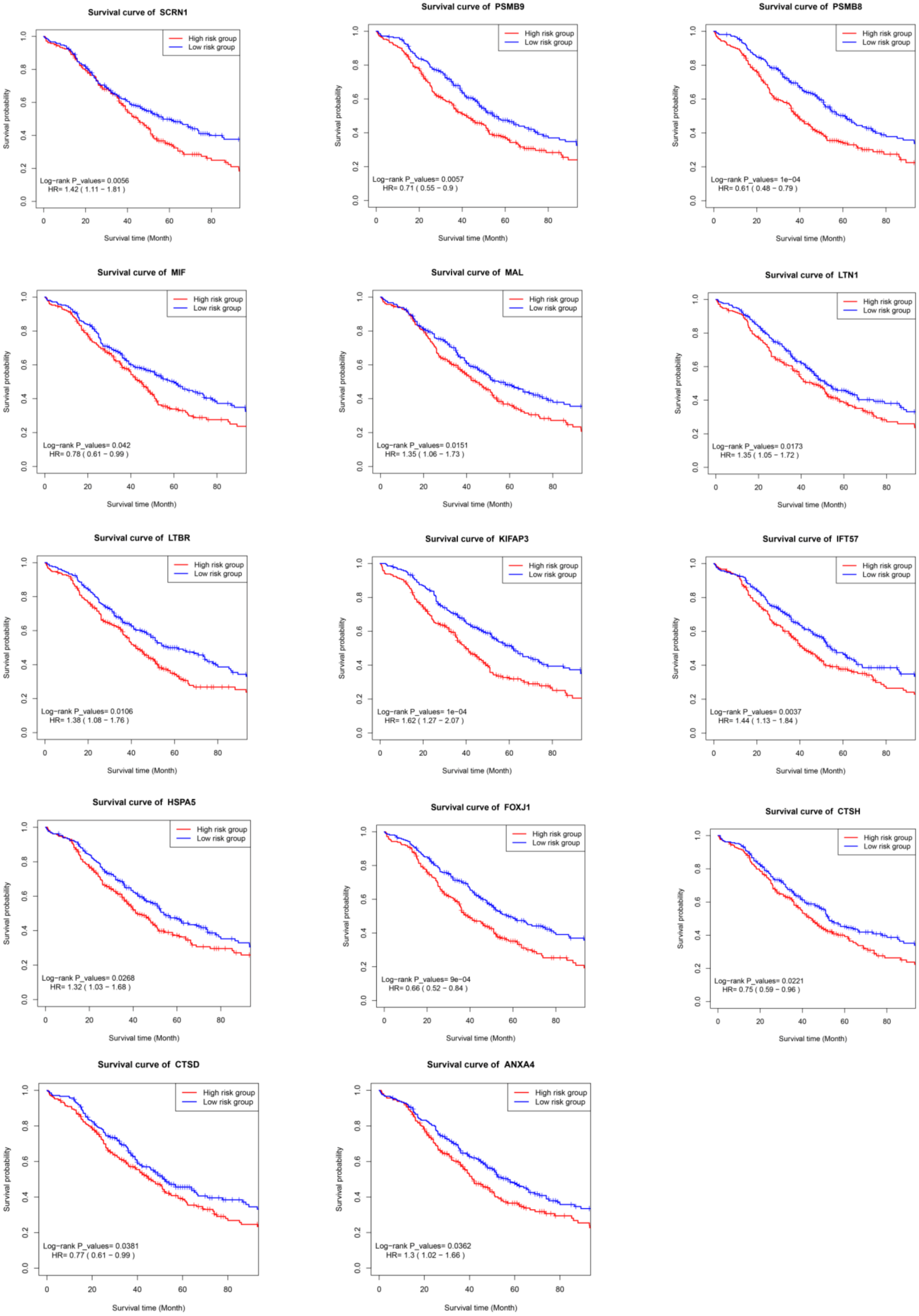

Supplementary Figure 2. Survival curves of immune genes

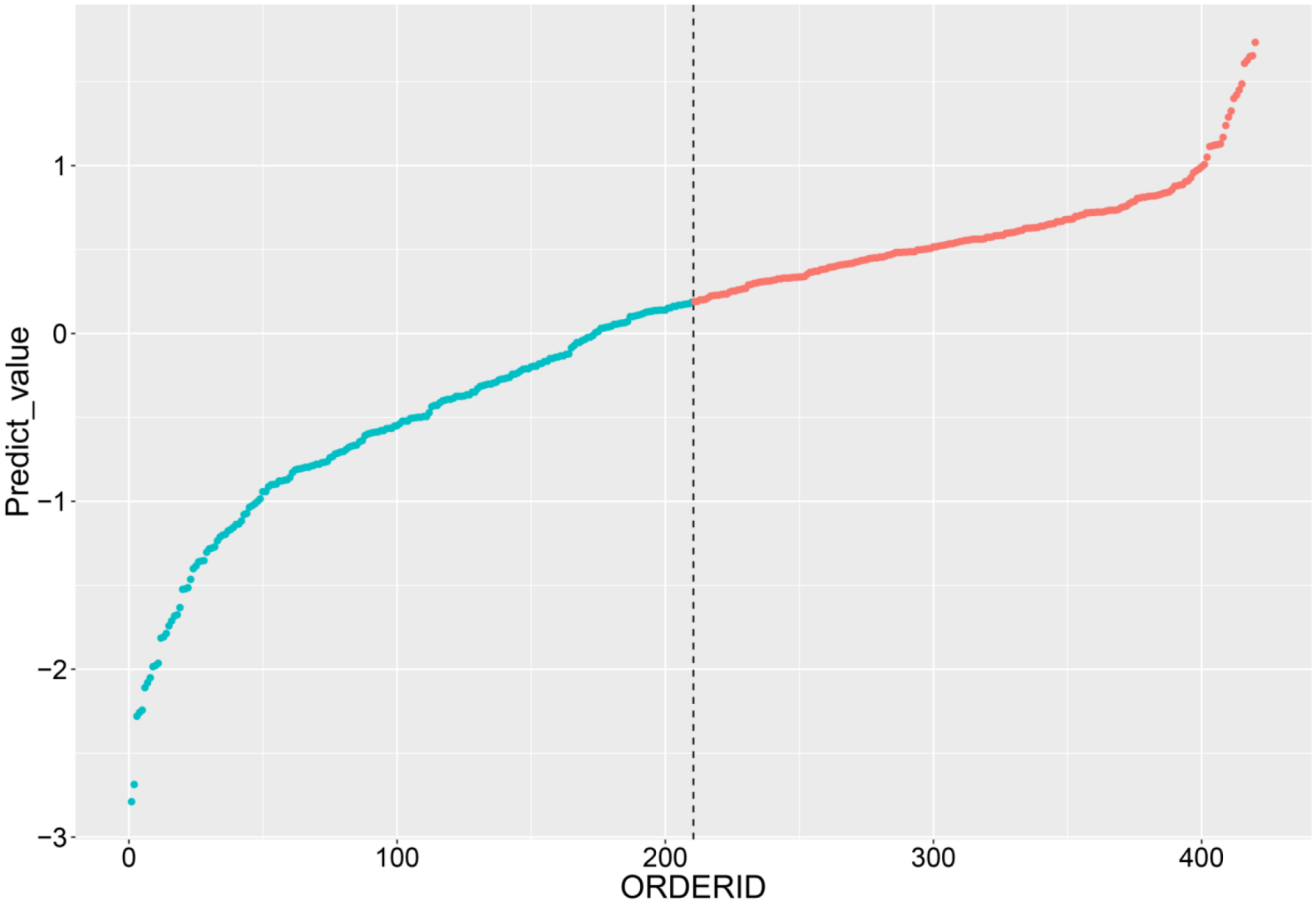

Supplementary Figure 3. Predictive value distribution chart

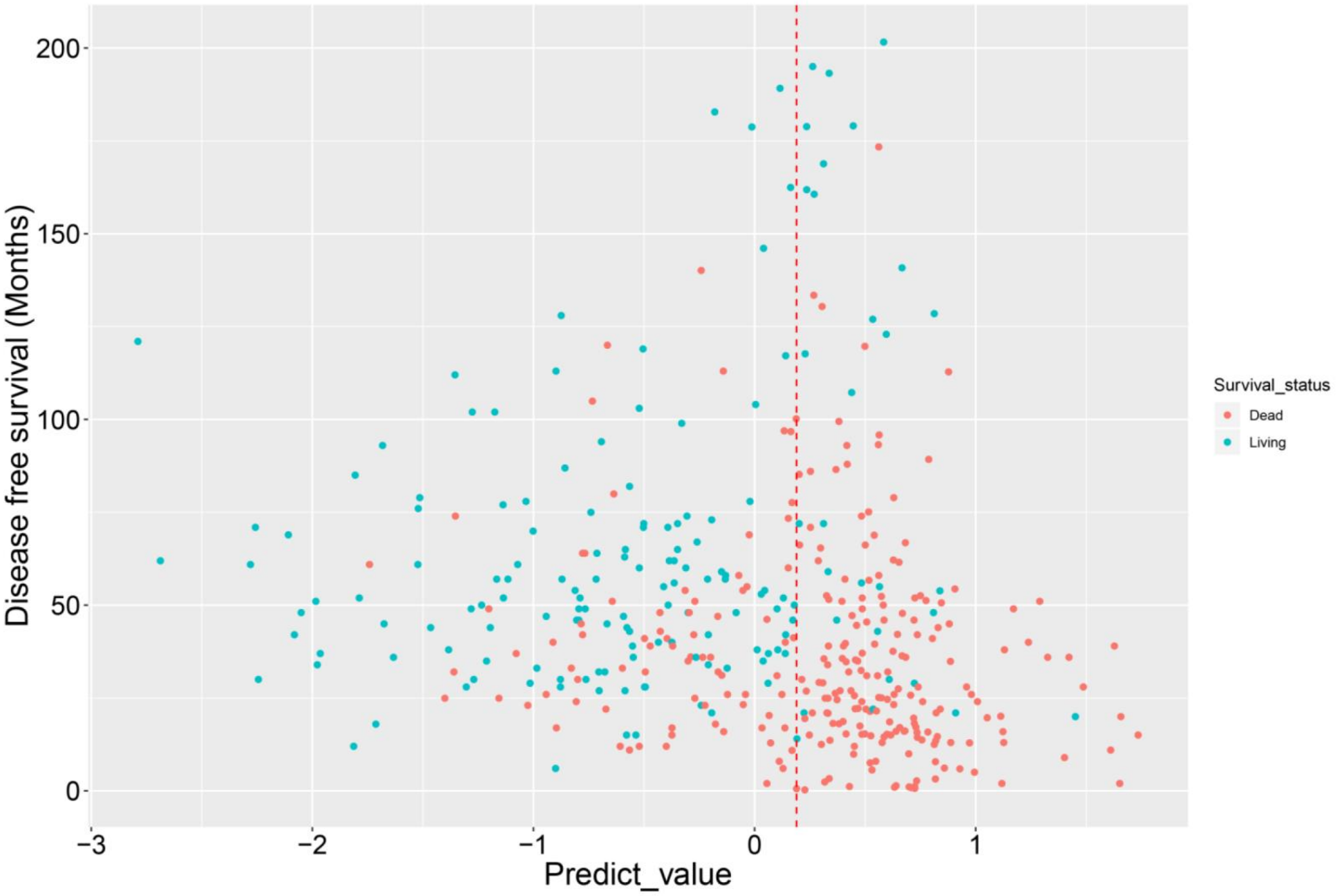

Supplementary Figure 4. Survival status scatter plot

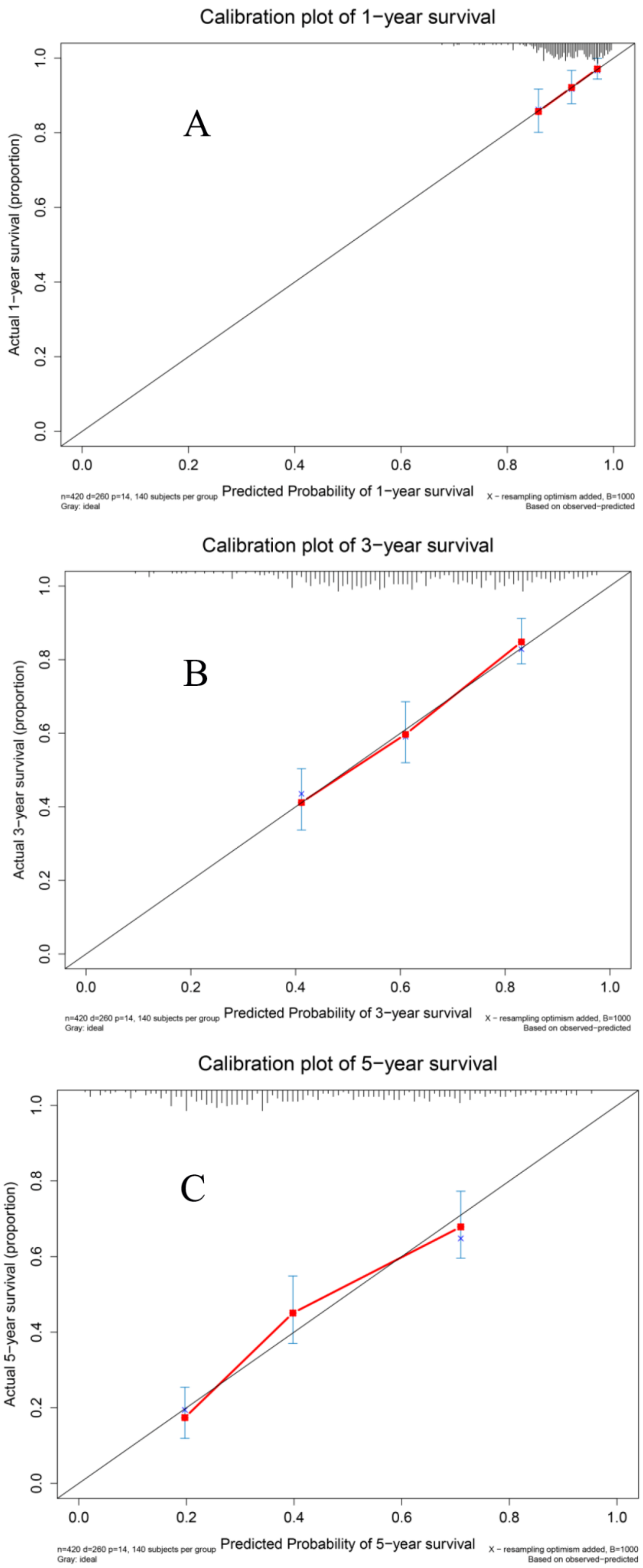

Supplementary Figure 5. Calibration curves for model cohort: (A) Calibration curve for 1-year survival; (B) Calibration curve for 3-year survival; (C) Calibration curve for 5-year survival

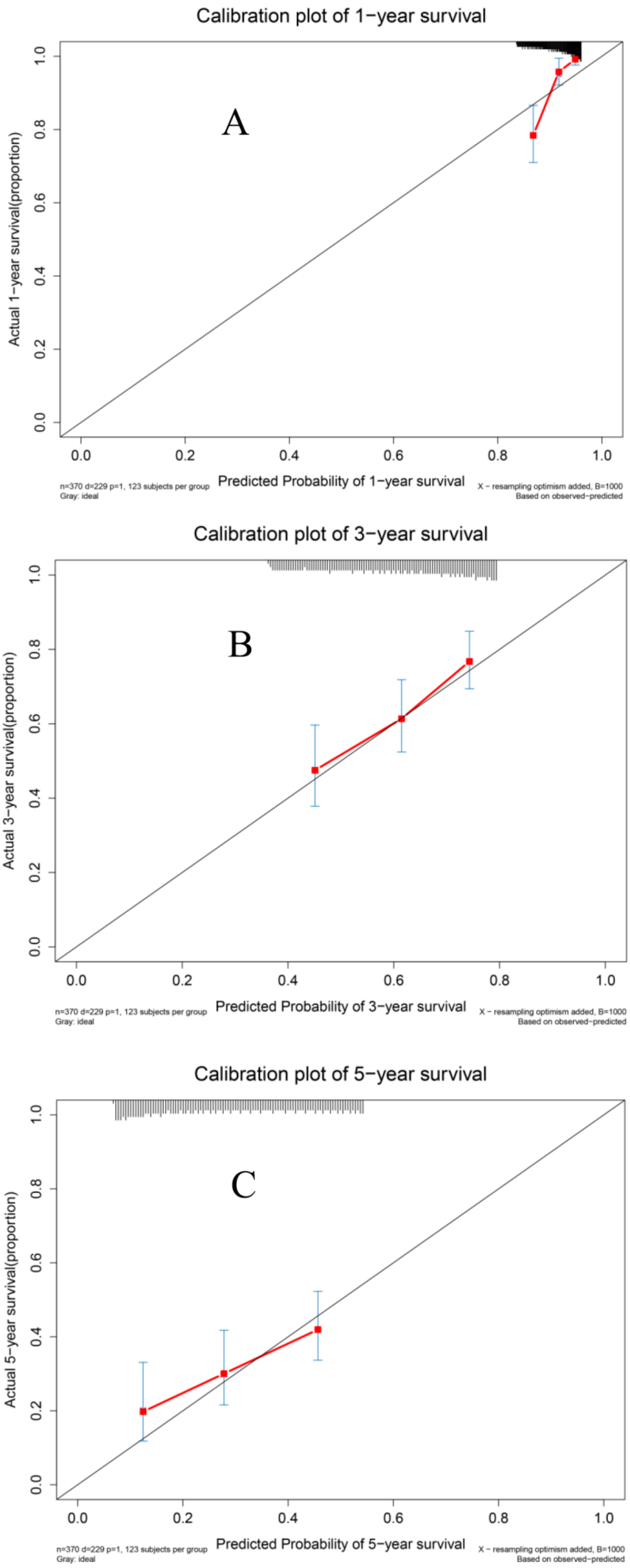

Supplementary Figure 6. Calibration curves for validation cohort: (A) Calibration curve for 1-year survival; (B) Calibration curve for 3-year survival; (C) Calibration curve for 5-year survival

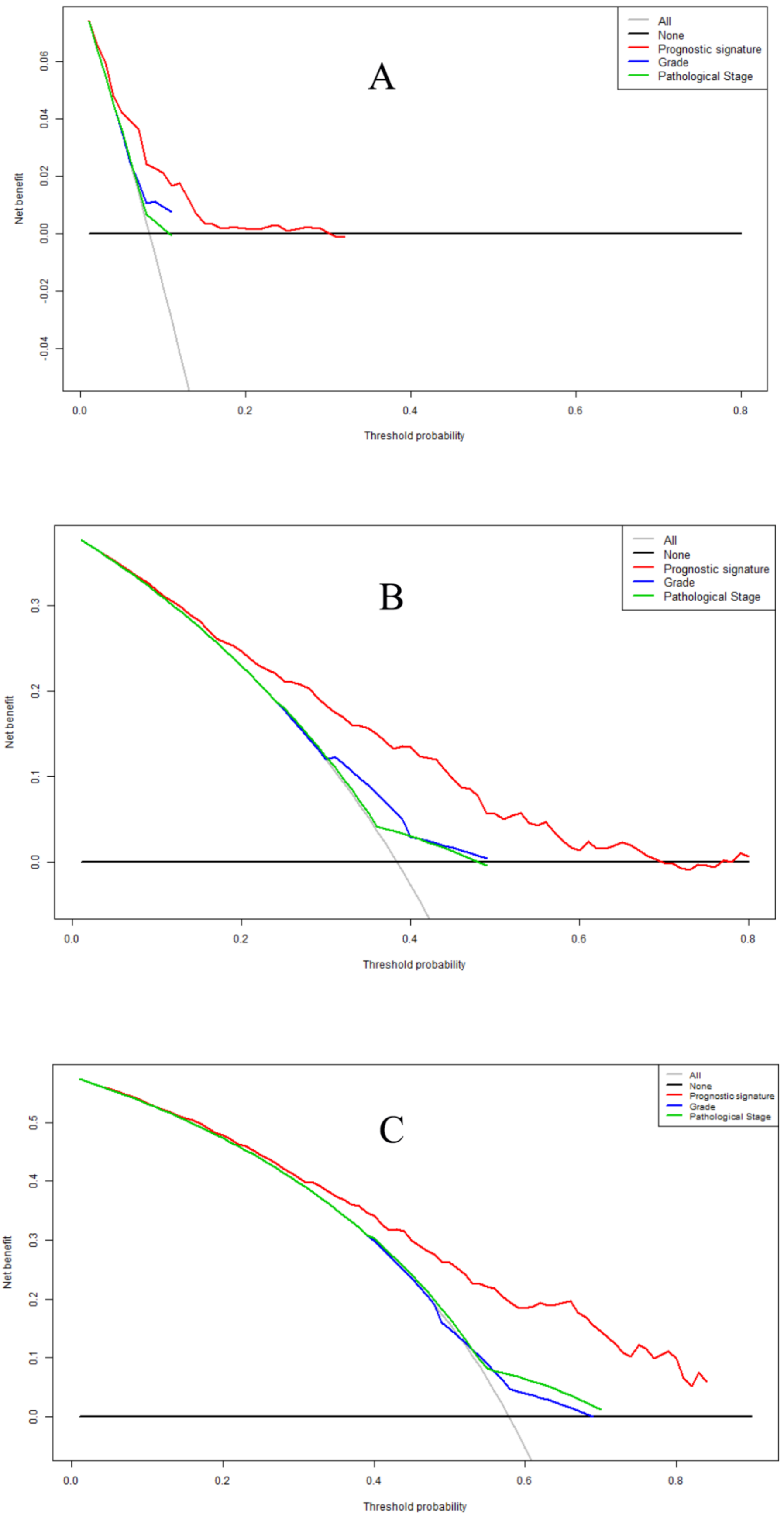

Supplementary Figure 7. Decision curves for prognostic model: (A) Decision curve for 1-year survival; (B) Decision curve for 3-year survival; (C) Decision curve for 5-year survival

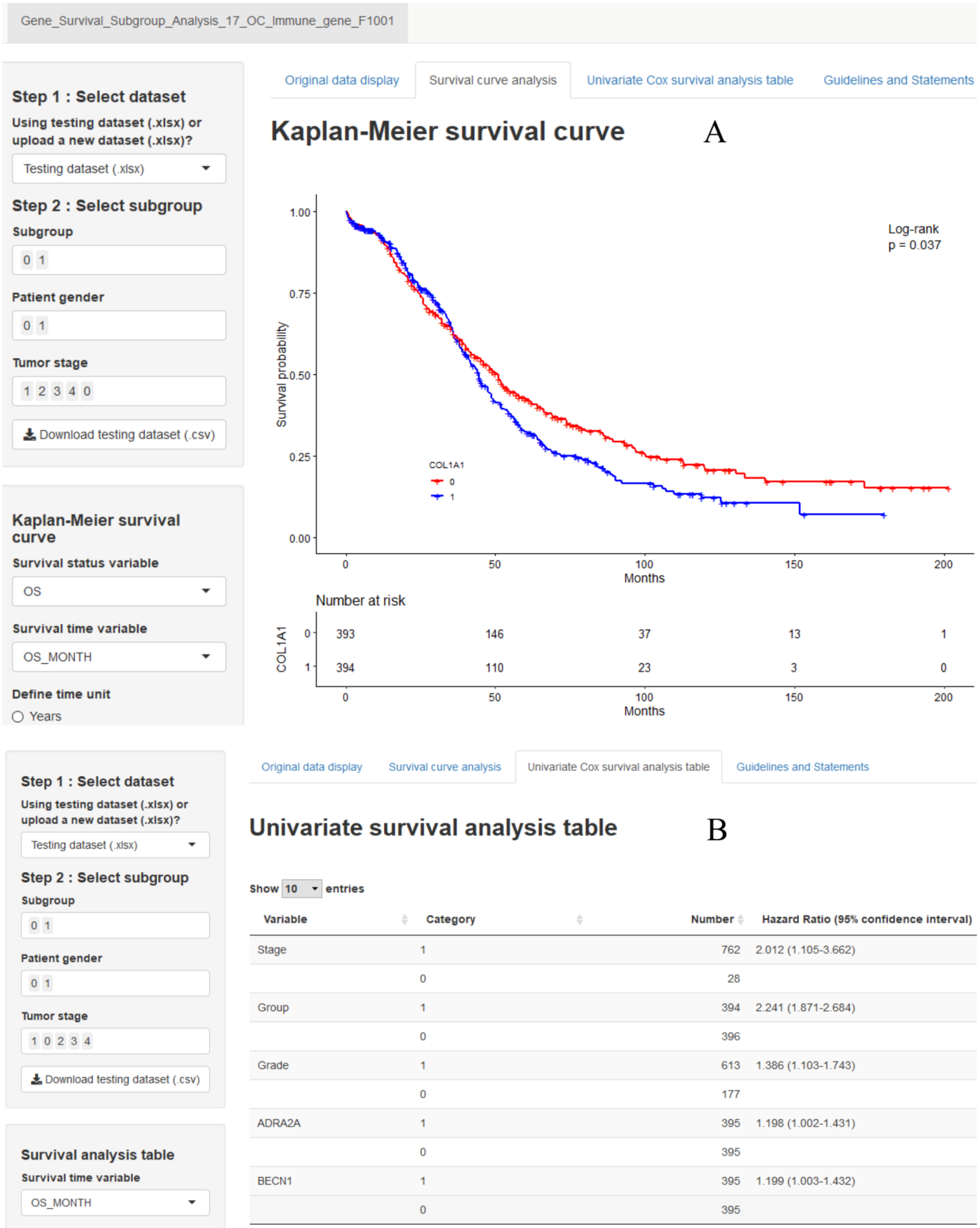

Supplementary Figure 8. Home page of Gene Survival Analysis Screen System: (A). Survival curves display page depicted and compared the survival curve between two defined subgroups; (B).Univariate survival analysis display page displayed results of univariate survival analysis for selected variables. Gene Survival Analysis Screen System allows users define different subgroups by themselves. Users are free to download, upload, and select the dataset for gene survival analysis.
